# Targeted Delivery System of Nanobiomaterials in Anticancer Therapy: From Cells to Clinics

**DOI:** 10.1155/2014/814208

**Published:** 2014-02-19

**Authors:** Su-Eon Jin, Hyo-Eon Jin, Soon-Sun Hong

**Affiliations:** ^1^Department of Drug Development, College of Medicine, Inha University, 3-ga, Sinheung dong, Jung-gu, Incheon 400-712, Republic of Korea; ^2^Department of Bioengineering, University of California, Berkeley and Physical Biosciences Division, Lawrence Berkeley National Laboratory, Berkeley, CA 94720, USA

## Abstract

Targeted delivery systems of nanobiomaterials are necessary to be developed for the diagnosis and treatment of cancer. Nanobiomaterials can be engineered to recognize cancer-specific receptors at the cellular levels and to deliver anticancer drugs into the diseased sites. In particular, nanobiomaterial-based nanocarriers, so-called nanoplatforms, are the design of the targeted delivery systems such as liposomes, polymeric nanoparticles/micelles, nanoconjugates, norganic materials, carbon-based nanobiomaterials, and bioinspired phage system, which are based on the nanosize of 1–100 nm in diameter. In this review, the design and the application of these nanoplatforms are discussed at the cellular levels as well as in the clinics. We believe that this review can offer recent advances in the targeted delivery systems of nanobiomaterials regarding *in vitro* and *in vivo* applications and the translation of nanobiomaterials to nanomedicine in anticancer therapy.

## 1. Introduction

Cancer is a worldwide disease with a leading cause of mortality, accounting for about 580,350 deaths, almost 1,600 people per day in 2013 from the statistical analysis of American Cancer Society in National Cancer Institute of the US [1]. About 1,660,290 of new cancer cases are also expected to be diagnosed in 2013. The 5-year relative survival rate is still somewhat low, at 68% for all cancers diagnosed between 2002 and 2008, although it has been up from 49% in the period from 1975 to 1977. For this reason, it is essential for targeted therapy for cancer to reduce adverse reactions and mortality rate and to save costs in clinical practice. Recently, targeted anticancer therapeutics such as monoclonal antibodies (mAbs) and tyrosine kinase inhibitors (TKIs) have been approved by the Food and Drug Administration (FDA) for the treatment of cancer [2]. The targeted therapy becomes an important element for the treatment of cancer as it helps to develop the anticancer therapeutics based on imaging and therapy (reducing the tumor size).

In this concept of targeted anticancer therapy, nanoplatforms are introduced with nanobiomaterial-based formulations or conjugation techniques in nanotechnology [3, 4]. Nanotechnology is a nanoscale-based technique in the fields of biomedical applications of pharmacology, bioengineering, biology, and medicine [5]. It currently relies on definitions provided by the National Nanotechnology Initiative (NNI) as follows [6]: (1) development of research and technology at the atomic, molecular, or macromolecular levels, within the scale of nanosize of approximately 1 to 100 nanometer in range, (2) devices and systems that have novel properties and functions based on the nanobiomaterials, because of their small and/or intermediate size, and (3) ability to control or manipulate at the atomic level. A variety of nanoparticular systems span the range from a few nanometers to hundreds of nanometers. When such nanoparticle-based systems are usually applied in solving the clinical problems, we often use the term “nanoplatforms” [7].

**Figure 1:**
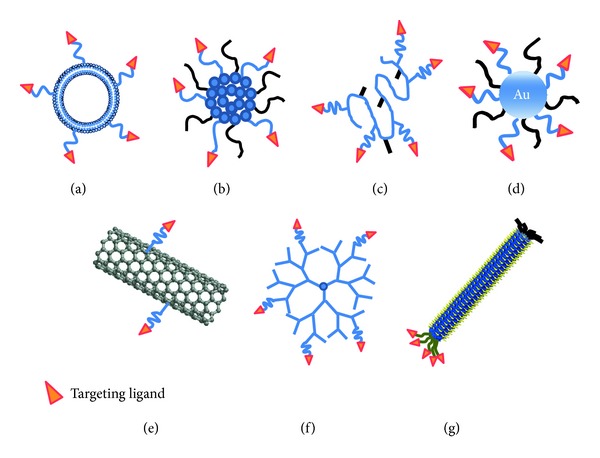
Schematic diagrams of the targeted delivery systems: (a) liposome, (b) polymeric micelle, (c) nanoconjugate, (d) gold nanoparticle, (e) carbon nanotube, (f) dendrimer, and (g) filamentous phage (M13, Fd).

Nanoplatforms have been developed to manufacture nanomedicines in preclinical and clinical studies for the administration of small molecules, genes, and peptides with improvement of given *in vivo* behavior [8–10].  Figure 1 shows the schematic diagrams of targeted delivery systems with nanoplatforms, such as liposome, polymeric micelle, nanoconjugate, gold nanoparticle, carbon nanotube, dendrimer, and phage-based nanoplatform. Several characteristics of an ideal tumor-targeted nanomedicine with nanoplatforms are presented for translational research of ideal anticancer therapeutics as follows: (1) increase of drug localization into the tumor by passive targeting or active targeting, (2) decrease of drug localization in sensitive, nontargeted, and normal tissues, (3) minimal drug leakage during transit to the target, (4) prevention of degradation and premature clearance of the drug, (5) retainment of the drug at the target site during the desired period, (6) facilitation of cellular uptake and intracellular trafficking, and (7) biocompatibility and biodegradability of nanoplatforms [10].  Table 1 illustrates the recently developed nanoplatforms for cancer targeting in preclinical studies. We introduce recent studies of targeted anticancer system in the part of “recently developed nanoplatforms for cancer targeting in preclinical studies.”

For successful translational research of nanoplatforms in anticancer therapy, Doxil is an FDA approved drug for the treatment of ovarian cancer, which is a long-acting pegylated liposomal formulation of doxorubicin [11, 12]. This product is based on a study of liposomal drug delivery systems for over four decades overcoming the limitations of anticancer drug and liposomes such as poor stability and reproducibility [13, 14]. It is one of the successful cases for the development of nanomedicines offering significant improvements over doxorubicin alone in clinical use. We deal with the translational research of nanomedicines in Table 2 and introduce the anticancer drugs approved by FDA in Table 3. Based on the ideal characteristics of nanoplatforms, they can be designed for the following: (1) sustained release of the drug, (2) passive accumulation of the tumor tissue, (3) ligand-based targeting of cell surface antigens or receptors with the modulation of endosomal uptake and membrane disruption, (4) drug release into the cytoplasm, and (5) protection from enzymatic degradation [15, 16]. Additionally, in this review, we demonstrate the applications of targeted delivery systems from the cells to the clinics for anticancer therapy, diagnostics, nanoimaging, bimodal imaging, and real-time intraoperative imaging.

## 2. Mechanism of Cancer Targeting by Nanoplatforms

**Figure 2:**
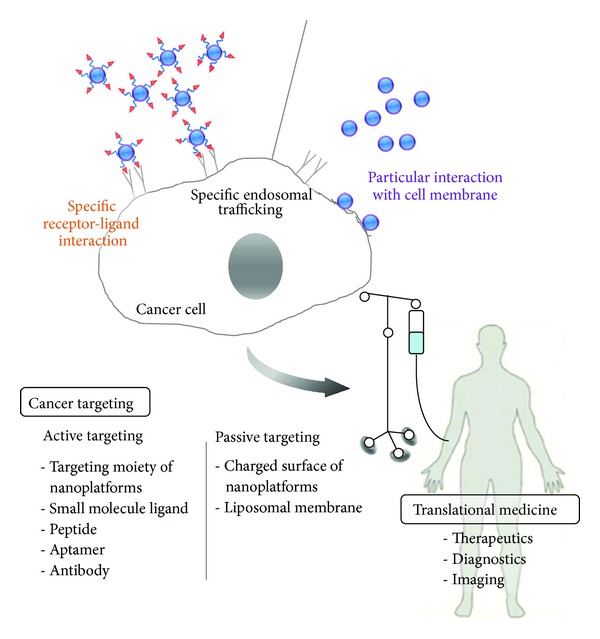
Overview of cancer targeting strategies from the cells to the clinics. This overview illustrates the most clinically relevant targeting strategies for anticancer therapy: passive targeting and active targeting. Passive targeting strategy is defined as the accumulation of nanoplatforms at the cancer cells by EPR effect, which uses nanoplatforms without targeting moieties. In this way, particular interaction will be proceeded to be internalized onto the cancer cells. On the other hand, active targeting strategy means the ligand-targeted or the receptor-mediated approach based on the cancer-specific targeting moieties of nanoplatforms, which interact with the specific receptor-ligand interaction on the cancer cells. Based on the cancer targeting strategies, translational medicine will be developed for the diagnostics, therapeutics, and imaging.

Many nanobiomaterial-based platforms have been designed and evaluated for drug targeting to cancers as shown in Figure 2. Most of these platforms are “passive targeting” concepts to improve the circulation time of the conjugated or encapsulated therapeutic drug such as liposomes, polymers, micelles, and nanoparticles. Solid tumors have blood vessels with enhanced vascular permeability and a lack of functional lymphatics, which allow extravasation of carrier materials with sizes of up to several hundreds of nanometers and are unable to eliminate extravasated nanomaterials. From the several reports, this EPR effect was shown even for the particles size of 1-2 *μ*m (e.g., *Lactobacillus* sp., *Salmonella* sp., etc.), which could make the particles accumulated in tumor tissues [17, 18]. Therefore, long-circulating nanomedicines are able to be accumulated in tumors over time based on a mechanism known as the enhanced permeability and retention (EPR) effect [19, 20]. The EPR effect is a unique phenomenon of solid tumors related to their anatomical and pathophysiological differences from normal tissues and is the most important strategy to improve the delivery of therapeutic agents to tumors for anticancer drug development. Examples of passively targeted nanoplatforms approved for clinical use are Doxil (Caelyx in Europe; pegylated liposomal doxorubicin), DaunoXome (nonpegylated liposomal daunorubicin), DepoCyt (nonpegylated liposomal cytarabine), Myocet (nonpegylated liposomal doxorubicin), Oncaspar (pegylated L-asparaginase), Abraxane (albumin-based paclitaxel), and Genexol-PM (paclitaxel-containing polymeric micelles, approved in Korea). In particular Doxil is a pegylated liposome-based, doxorubicin-loaded anticancer drug, which is FDA approved for the treatment of ovarian cancer as mentioned above. This drug has a severe cardiotoxicity although it overcomes poor stability and reproducibility of liposomes and improves a biodistribution of doxorubicin in tumor tissues by EPR effect. In addition, Abraxane is a Cremophor EL-free albumin-bound paclitaxel. It reduced Cremophor EL-associated side effects of Taxol such as severe anaphylactoid hypersensitivity reactions, hyperlipidaemia, abnormal lipoprotein patterns, aggregation of erythrocytes, and peripheral neuropathy [21]. Next-generation nanomedicines for anticancer therapy are developing alternative approaches with recently developed nanoplatforms minimizing side effects of surfactants in formulations and targeting tumor tissues. Several additional passively tumor-targeted nanomedicines are currently in clinical trials (Table 2), and many other ones are in early and late-stage preclinical development [9, 22–25].

On the other hand, “active targeting” strategy indicates the use of targeting ligands like antibodies and peptides which are attached to drugs and drug delivery nanoplatforms for binding to receptor expressed at the target site. In this strategy, active targeting systems with ligands and antibodies need to be accumulated first in tumor tissues by EPR effect, and then active targeting could be achieved. Targeting ligands for actively targeting nanomedicines are improving cellular internalization via endocytosis-prone surface receptors, such as folate [26], galactosamine [27], EGF [28], and transferrin [29]. To date, targeting techniques for active targeting are advanced at the preclinical level, however, only antibody-based nanomedicines have been approved for clinical use (Tables 2 and 3). Zevalin (CD20-targeted ^90^Yttrium-ibritumomab tiuxetan), Bexxar (CD20-targeted iodine-131 tositumomab), Ontak (CD25-targeted diphtheria toxin-IL-2 fusion protein), and Mylotarg (CD33-targetd gemtuzumab oxogamicin) have been successfully used for non-Hodgkin's lymphoma, T-cell lymphoma, and acute myeloid leukemia. In addition, it has necessitated the use of peptides to enable the cell internalization of cancer drugs such as cell-penetrating peptides, protein-transduction domains, oligoarginine, and TAT [30].

Active targeting to receptors, which are overexpressed by angiogenic endothelial cells, can reduce blood supply to tumors that deprive the tumor cells from oxygen and nutrients in solid tumors. Ligands used for drugs and drug delivery platforms to tumor vasculature include antibody fragment L19 [31], as well as several cyclic and linear derivatives of the oligopeptide RGD and NGR, which bind to angiogenic endothelium through the integrins (*α*_2b_ *β*_3_, *α*_v_ *β*_3_, and *α*_5_ *β*_1_) and aminopeptidase-N (CD13), respectively [32, 33].

Although active targeting systems are extensively studied to develop anticancer therapeutics, there has been a less example of clinical use compared to passive targeting systems. Discussing these problems to overcome, active targeted nanoplatforms are physicochemically unstable for blood circulation in the body and hard to be accumulated in tumor tissues due to their size of conjugated targeting ligands of formulations (e.g., monoclonal antibodies) [34]. In addition, active targeting systems can be eliminated by nanoparticular surface opsonization (nonspecific protein adsorption) and nanoparticular uptake and retention to reticuloendothelial systems resulting in poor efficiency of active targeting system *in vivo* [35]. Based on these fundamental problems and technical barriers, multifunctionality, targetability, and stability are necessary to overcome the low efficiency of active targeting nanoplatforms.

## 3. Nanoplatforms of Anticancer Therapeutics and Application in Clinical Trials

Targeted delivery systems of nanobiomaterials are currently in the process of developing nanoscale-sized platforms or surface modification of nanoplatforms with active targeting ligands that support multifunctionality due to poor solubility of anticancer drugs or toxic effect of drugs on noncancerous cells and tissues [36, 37]. Most of the anticancer drugs are poorly soluble or insoluble in water. Thus, organic solvents or toxic surfactants are usually applied for the formulation of anticancer therapeutics even used in clinics. In addition, anticancer drug alone has a toxic effect on normal cells and tissues without target specificity [38, 39]. Therefore, several nanoplatforms in 100 nm diameter based on nanobiomaterials have been used to administer the anticancer drugs to minimize the adverse toxicity and to maximize the drug effect within the therapeutic index [40].

Nanoplatforms offer solubility of poorly soluble anticancer drugs in water preparing soluble suspensions with the minimum need for organic solvents and surfactants which cause toxicity [40, 41]. These platforms are accumulated in the tumor tissues by the EPR effect, as mentioned above. The nanoplatforms are also used in the targeted delivery systems of active targeting using the decoration with receptor-targeted ligands or tumor antigen sensitive antibodies together with anticancer drugs [22, 37, 42–44]. The anticancer drugs encapsulated into the nanoplatforms, specifically liposomal nanoplatforms and polymer-based nanoplatforms, are undergoing clinical trials [43, 45, 46]. Table 2 shows the nanomedicines of anticancer drugs in clinical trials.

### 3.1. Doxorubicin

Doxorubicin, a very effective anticancer drug, is widely used in the treatment of breast, ovarian, bladder, and lung cancers [47]. Mechanism of action of doxorubicin is the blocker of topoisomerase II, which is an important enzyme in the DNA replication process that unwinds the DNA helix. The mechanisms of doxorubicin also include DNA cross-link and ROS generation besides inhibiting topoisomerase II. Based on these mechanisms of action, doxorubicin has a potent antitumor activity in tumor cells inducing cell death. However, doxorubicin is associated with the severe side effects on the heart including irreversible myocardiotoxicity and fatal congestive heart failure [48]. To decrease the toxicity of doxorubicin, nanoplatforms were introduced to enhance pharmacokinetic parameters with an accumulation of drugs in the tumor tissues, thereby minimizing the cardiotoxicity. Liposomes and polymeric nanoplatforms were studied and developed as nanomedicines via an intravenous route. Myocet, MCC-465, MM-302, SP1049C, and NK911 are the liposomal or polymeric nanopaltforms of doxorubicin in the clinical trials [49]. Myocet, MCC-465, and MM-302 are based on the liposomal nanoplatforms at the range of 100–140 nm in diameter [50]. In the cases of MCC-465 and MM-302, the target-specific ligands of F (ab′)_2_ fragment in human mAb GAH or tumor-specific antigen and scFv/ErbB2 (HER2) are incorporated into the liposomal nanoplatforms, respectively. In other words, SP1049C and NK911 are used in the polymeric micelles. SP1049C is a Pluronic-based micellar formulation of doxorubicin [51] and NK911 is a core-shell-type polymeric micellar nanoplatform of doxorubicin which consists of a block copolymer of PEG (m.w., 5000) and poly(aspartic acid) (30 units) conjugated with doxorubicin [52]. Doxorubicin is entrapped into the highly hydrophobic inner core of the polymeric micellar nanoplatforms.

### 3.2. Paclitaxel

Paclitaxel is a chemotherapeutic agent for the ovarian, breast, and lung cancers as well as Kaposi's sarcoma [53]. It is a mitotic inhibitor with a stabilizing activity of the microtubule assembly interfering the normal breakdown of microtubules during cell division. Paclitaxel was originally extracted from the Pacific yew tree, *Taxus brevifolia*. Bristol-Myers Squibb commercially developed paclitaxel, a famous trademark, Taxol. However, paclitaxel itself has severe adverse responses such as peripheral sensory neuropathy [54, 55], anaphylaxis, and hypersensitivity reactions due to its solubilizing materials (Cremophor EL and ethanol) [55]. Therefore, the development of nanoplatforms is essential to overcome these problems of formulation for the improvement of pharmacokinetic parameters and the toxic adverse reactions in the normal tissues [56]. EndoTAG-1 [57], LEP-ETU [58], Genexol-PM [59], NK105 [60], and Opaxio [61] are developed as paclitaxel-loaded nanoplatforms undergoing clinical trials. EndoTAG-1 and LEP-ETU are based on the liposomal nanoplatforms. In particular, EndoTAG-1 is based on the cationic liposomal formulation, which can interact with the newly developed and negatively charged endothelial cells in the disease states for the growth of tumor blood vessels targeting the blood supply to the tumor cells [57]. Polymeric nanoplatform-based paclitaxel formulations are also developed such as Genexol-PM, NK105, and Opaxio. Paclitaxel is conjugated to poly(l-glutamic acid) (PGA) in Opaxio [61], monomethoxy poly(ethylene glycol)-block-poly(D,L-lactide) (mPEG-PDLL) in Genexol-PM [59], and NK105 polymers of PEG as the hydrophilic segment and modified polyaspartate as the hydrophobic segment in NK105 [62], respectively.

### 3.3. Platinum-Based Anticancer Drugs

Cisplatin (cisplatinum or *cis*-diamminedichloroplatinum (II)) is also used in chemotherapy, which is a platinum-based anticancer drug for the treatment of various cancers including sarcomas, some carcinomas (e.g., small cell lung cancer and ovarian cancer), lymphomas, and germ cell tumors [63, 64]. Mechanism of action in platinum-based anticancer drug is DNA cross-linking to interfere with the cell division by mitosis triggering apoptosis or cell death. Oxaliplatin and carboplatin are also included in platinum-based anticancer therapeutics. In clinical trials, oxaliplatin (MBP-426) [65] and cisplatin (NC-6004, Nanoplatin) [66] are studied for their applicability to advanced/metastatic solid tumors. In particular, Nanoplatin in combination with gemcitabine is evaluated for the treatment of advanced/metastatic pancreatic cancers. MBP-426 is reported as an oxaliplatin-based liposomal nanoformulation with the surface modification of transferrin targeting transferrin receptors in disease states. In the case of polymeric nanoplatforms, Nanoplatin, a polymeric micelle-based cisplatin of 30 nm in diameter, is studied for its kidney toxicity reduction capabilities compared to cisplatin alone.

### 3.4. Camptothecins

Camptothecin and irinotecan, a water-soluble derivative of camptothecin, are cytotoxic alkaloids isolated from *Camptotheca acuminate* [67]. The target of these camptothecins and their derivatives is topoisomerase I to inhibit the replication in the cells. They bind to the topoisomerase I and DNA complex generating a stabilized ternary complex to prevent DNA religation and to cause DNA damage resulting in apoptosis. Camptothecin and its derivative are limitedly used due to lipophilicity and instability of the lactone ring structure by hydrolysis despite their superior anticancer activity [68]. Therefore, the nanoplatform-based camptothecin (S-CKD-602 [69] and CRLX101 [70]) and irinotecan (SN-38, NK012 [71]) are developed undergoing clinical trials. S-CKD-602 and CRLX101 are used in the nanoplatforms of a pegylated liposome and a polymeric micelle, respectively. In the case of irinotecan (SN-38, NK012), it is a polymeric micelle-based active metabolite of camptothecin to exploit the EPR effect in the diameter size of 20 nm. This system is constructed in an amphiphilic block copolymer, PEG-PGlu (SN-38), by the self-assembly in the aqueous media. NK012 had an antitumor activity with tolerance that included partial responses and several occurrences of prolonged stable disease across a variety of advanced refractory cancers in the clinical studies.

### 3.5. Gene Therapy

Plasmid DNA and siRNA for cancer gene therapy are also used for the treatment of cancers in the clinical trials. The aim of gene therapy is to kill the cancer cells blocking the transduction of the tumor cells or inhibiting the disease-induced proteins without any damage to the normal cells, using cancer-specific genetic materials [72]. For the current clinical trials, p53 gene (SGT53-01) [73], RB94 plasmid DNA (SGT-94) [74], and RRM2 siRNA (CALAA-01) [75] are studied for the treatment of solid tumors. These materials are based on tumor-specific nanoplatforms, which are the liposomal (SGT53-01 and SGT-94) or cyclodextrin-based nanoparticular (CALAA-01) systems decorated with a target ligand of transferrin receptors in the tumor cells. These nanoplatforms are necessary in delivering the genetic materials into the tumor cells due to the instability in the biological fluids (e.g., enzymatic digestion) and the hydrophilicity of genetic materials (e.g., poor uptake to cells).

## 4. Recently Developed Nanoplatforms for Cancer Targeting in Preclinical Studies

### 4.1. Liposomes

Liposomes have been developed as bioinspired nanoplatforms, which define the phospholipid bilayer showing the properties of the cell membrane [76, 77]. The liposomal nanoplatform can carry the hydrophilic drug at the aqueous core inside the bilayer and the hydrophobic drug at the lipid membrane. This nanoplatform has a lot of advantages for the delivery system with a great attention given their biocompatibility and targetability from the *in vitro* characterization to translational research [50, 78, 79]. For cancer targeting, liposomes are extensively used as passive targeting agents and ligand-mediated or stimuli-sensitive targeting agents (active targeting agents) [80]. Based on their biocompatibility, liposomes have been early developed as nanoplatforms to transfer small molecules in the tumor tissues [14]. Various anticancer drugs such as doxorubicin, doxorubicin derivatives, paclitaxel, and platinum-based anticancer drugs are usually loaded into the liposomal platforms (Table 1). These are 100–200 nm in average size, which makes liposomes take a trip into the tumor tissues.

Liposomes can also be used in the targeted delivery system with several modification of the liposomal surface design [81–84]. Cancer targeting systems are rapidly used in broad applications for the tumor-specific ligands [85, 86] or tumor-associated antigens [87] although liposomes are possible to be used in anticancer therapy as the delivery carriers for passive cancer targeting. The cancer-targeted therapy with targeting ligands includes leukocyte differentiation antigen (CD33) for acute myeloid leukemia [88], GD2 for neuroblastoma [89], and the folate receptor for wide human tumors [90, 91]. In addition, integrins [92], vascular endothelial growth factor receptors (VEGFR) [93], and CD13/aminopeptidase N [94] are also used as the targeting ligands. Liposomes with cyclic Asn-Gly-Arg (cNGR) peptide targeted to CD13 were currently formulated with lysogenic lipids-loaded doxorubicin for the potential treatment of human fibrosarcoma [95]. These systems are not only ligand-targeted systems for cancer metastasis, but also the hyperthermia-targeted systems for tumor hyperthermia, which regulates the release of doxorubicin specifically in the hyperthermal tissues for cancer targeting.

Liposomes can also be used as one of the components in the nanohybrid systems [96]. A study by Von Maltzahn et al. reported a complexed nanohybrid system for the treatment of human breast cancer in the xenograft tumor model of MDA-MB-435 cells [97]. This system is the complex of “signaling modules” to activate and broadcast the tumor location and “receiving modules” to carry the nanoplatform-based diagnostic agents and therapeutic drugs. They used the doxorubicin-loaded liposomes as therapeutic cargos with iron oxide nanoworms as the diagnostic agents in circulation. In addition, for the activation of doxorubicin-loaded liposomal cargos, PEG-decorated gold nanorods and tumor-targeted truncated tissue factor proteins (tTF-RGD) were previously introduced as the signaling modules to induce the heat specifically directed coagulation on binding to the angiogenic receptors in tumors before adding the doxorubicin-loaded liposomes. This system can be applied for the targeted theranostic agents to detect and treat the tumors together. Liposomes can also be used together with carbon nanotubes [98, 99]. Huang et al. reported carbon nanotubes encapsulated into the liposomes, which carried paclitaxel and anti-ErBb2 (Her2) mAb for this system SK-BR-3 and BT-20 breast cancer cells and checked the *in vitro* cytotoxicity [98]. This system can also be applied to theranostics for the potentiality of diagnostics and therapeutics.

### 4.2. Polymeric Nanoparticles/Micelles

Polymers are innovative nanobiomaterials to be engineered for the delivery system of drugs, genes, and peptides [41, 100]. Polymeric nanoparticles or micelles are easily prepared to be nanosized with various designs, which are relatively smaller in size compared to lipid-based formulations that generally range in size of 1–50 nm. Polymers can be used to carry multiple ligands for cancer targeting and imaging molecules for cancer diagnostics with a simple conjugation-based structural modification [101]. Among the polymers, PEG and poly(lactic-co-glycolic) acid (PLGA) are extensively used as biocompatible polymers for translational medicine [102, 103].

PLGA is one of the most widely used polymers due to its biocompatibility, which is degraded to the monomers of lactic acid and glycolic acid in the body [104]. PLGA was approved by US FDA and European Medicine Agency (EMA). PLGA-based nanoparticles are successfully applied for drug delivery of the biomedical approaches for the development of translational medicine [105]. For example, paclitaxel-encapsulated PLGA nanoparticles were formulated with a strong enhancement of the cytotoxic effect of paclitaxel on the tumor cells *in vitro* and *in vivo* compared to commercial formulation, Taxol [106, 107]. Hasan et al. published the cationic lipid-coated PLGA nanoparticles with a unique soft lithography particle molding process, which was applied for the delivery of siRNA in the prostate cancer cells [108]. In this study, they coated the PLGA nanoparticles with cationic lipid for a successful internalization to the cells based on a charge-charge interaction of the cell membrane and nanoparticles because PLGA has a negative charge. Based on the addition of targeting ligands like cLABL (ICAM-1 targeting) [109], folate (folate receptor) [110, 111], prostate-specific receptor antigen (prostate-specific receptor targeting) [112, 113], RGD (integrins *α*_v_ *β*_3_) [114, 115], and AS1411 (nucleolin targeting) [116, 117], PLGA nanoparticles have the potential to improve drug efficacy via target specificity of nanoplatforms *in vitro* and *in vivo*.

Although these nanoparticles have an effective function in cancer therapy in preclinical trials, these nanoparticles can be removed in circulation based on the biological barrier and reticuloendothelial system (RES), which performs an opsonization to macrophages that are internalized by phagocytosis [118]. PEG is the most commonly used polymeric moiety for the surface modification of nanoplatforms [119]. Nanoparticles can be usually decorated with targeting ligands conjugated with PEG. PEG originally has a function to make nanoparticles sterically stable for the prolonged circulation in the blood after administration. This method is called “pegylation” [120]. It demonstrates the hydrophilic moiety of PEG on the particular surface in the nanoplatforms, which provides the steric hindrance of a particular system to be shielded against the RES system for a prolonged delivery of the drug. Aravind et al. studied the long-circulating, drug-loaded polymeric micelles enhancing tumor permeability with a TGF*β* inhibitor in the poorly permeable pancreatic tumors in a murine model of the C26 or BxPC3 tumors [116]. They loaded 1,2-diaminocyclohexane-platinum(II)(DACHPt), which is the parent complex of oxaliplatin, in the polymeric micelles of PEG-b-poly(glutamic acid) (PEG-b-P(Glu)) copolymer and P(Glu) homopolymer. After the size-based screening of permeability into the tumors, 30 nm sized micelles had a relatively enhanced tumor permeability, when transforming growth factor *β* was administrated together with the micelles.

### 4.3. Nanoconjugates

Nanoconjugates have extensively been studied as the smart nanoplatforms with active functional groups to prepare the covalent binding for the anticancer therapeutics [121]. Polymeric nanoconjugates are generally synthesized with a simple conjugation of the functional groups in the polymer such as –OH, –COOH, and –NH_2_. These steps are stepwise reactions, which need to avoid uncontrollable side chemical synthesis. Nanoconjugates include the nanoplatforms of polymeric nanoparticles/micelles and other nanoparticles based on the characteristics of conjugation technique to prepare the nanoconjugate platforms using the functional groups of nanobiomaterials. Polymer-drug conjugates [122, 123] or mAb conjugates [42] were reported for targeted anticancer therapy. Xiong et al. introduced the cisplatin-based poly(*γ*, l-glutamic acid)-citric acid-based nanoconjugates [123], and Segal and Satchi-Fainaro illustrated the polymeric nanoconjugate-based therapeutics with favorable polymers such as N-(2-hydroxypropyl)methacrylamide (HPMA), polyglutamic acid (PGA), and *β*-poly(L-malic acid) (Polycefin) [122]. In addition, Julien et al. showed mAb-targeted nanobiomaterials for anticancer therapy [42]. They described the mAb-drug conjugates with an introduction of mAb-based therapeutics such as CD20 (Rituxan for B-cell lymphoma), HER2 (Herceptin for breast cancer), VEGF (Avastin for colon, lung, breast, and renal cancer), and EGFR (Erbitux for colon and lung cancer).

These platforms have more advantages than other nanoplatforms, like micelles and liposomes, in the way that they are small in size and chemically stable in biological fluid. For this reason, these platforms are easily applied to the functionalization of multiple-target ligands or theranostic agents with polymers, peptides, proteins, or other nanoparticles. For example, folate is a widely used ligand for cancer targeting, which can have an interaction with the overexpressed folate receptors in cancer states [124]. Folate receptors are highly overexpressed in epithelial, ovarian, cervical, breast, lung, kidney, colorectal, and brain tumors while they are restrictedly expressed in the normal tissues, such as the lung, kidney, placenta, and choroid plexus, which are limited to the apical surface of the polarized epithelia [124, 125]. Folate has a lot of advantages to be used for a targeting ligand through the conjugation to nanobiomaterials based on its small molecular weight (441 Da) and the easy preparation method of folate-linked nanobiomaterials due to the stability of folate over a broad range of temperatures and pH values [126]. Zwicke et al. focused on the folate-based nanoconjugates for anticancer therapy with doxorubicin or paclitaxel and cancer imaging with other contrast agents like gold nanoparticles, iron oxide nanoparticles, or carbon nanotubes [127].

The nanoconjugate systems can be the effective targeting nanoplatforms of macromolecules to the tumor tissues based on their ultrasmall size although they carried the ligand-based targeting systems of cell surface antigens, peptides, or polymers. The albumin-based nanoconjugates were introduced by Ming et al. [128], which carried phosphorodiamidate morpholino oligomer (PMO) type splice-switching oligonucleotides (SSOs) with a RGD peptide for integrin *α*_v_ *β*_3_, a cell surface glycoprotein, as an active targeting ligand and a fluorescence label. This system was very small based on 13 nm of size. It had a high specificity without cytotoxicity. In particular, it was applied to the tumor spheroids of A375 cells to check the uptake and penetration of the albumin-based nanoconjugates into the three-dimensional (3D) cultures. In addition, these nanoconjugates can overcome the drawbacks of conventional chemotherapy such as drug toxicity to normal cells and cancer drug resistance by specific targeting and activating the cancer cells via the multiple decorations of target ligands on the nanoconjugates. Mittapalli et al. also reported the paclitaxel-ultrasmall hyaluronic acid (HA) nanoconjugates [129], which is a CD44 receptor targeting system for the treatment of brain metastasis of breast cancer in a model of MDA-MB-231 breast cancer cells. They used an ultrasmall HA (3–5 kDa) as a target ligand to interact with CD44 receptors on the surface of the cancer cells. In addition, this ultrasmall HA-mediated cellular uptake of paclitaxel avoided the P-glycoprotein-mediated efflux showing the drug resistance in cancer cells. In particular, this system exhibited a small size of 2-3 nm, like a single molecule of the nanoconjugates, and can self-assemble into larger particles.

### 4.4. Inorganic Nanoparticles—Iron Oxide Nanoparticles, Superparamagnetic Iron Oxide Nanoparticles, and Gold Nanoparticles

Inorganic nanoparticles have been currently investigated as contrast agents in clinical practice [130]. Iron oxide nanoparticles and superparamagnetic iron oxide nanoparticles have been studied extensively as contrast agents because they enhance the negative contrasts and give us darker images of the interest regions in magnetic resonance imaging (MRI). Iron oxide nanoparticles have a magnetic moment to be changed by an ambient thermal energy. This system has been widely used for MRI contrast enhancements, as well as tissue-specific release of therapeutic agents [131]. Superparamagnetic iron oxide (SPIO) nanoparticles are either form which includes the inside of the core of magnetic nanoparticles with a polymeric coating or a homogeneous integration into the polymeric nanoparticles [4]. This nanoparticular system has a small size with the range of 3–6 nm of the core size and 20–150 nm after dextran coating such as Feridex and Combidex, which is a superior biocompatible magnetic material-based biomedical technique with respect to other magnetic materials, both based on oxides or pure metals [132]. In addition, this system can deliver anticancer drugs such as doxorubicin and methotrexate, which is used as theranostic cargo system due to its small size [133, 134]. In addition, the gold nanoparticles were used together with the iron oxide nanoparticles to study the MRI contrast agents, as well as the optical probes exploiting the reflectance signal of the gold nanoparticles [135].

The gold nanoparticles can also be used in an ultrasensitive assay technique to detect cancers [136]. Peng et al. introduced the functionalized gold nanoparticles in combination with the pattern recognition methods to diagnose the lung cancer from breath testing [137]. This method is an *in vitro *sensor array technique of the detection of biomarkers in exhaled breath of lung cancer patients as a noninvasive diagnostic tool. In addition, Thaxton et al. reported the combination system based on the magnetic microparticles and the gold nanoparticles conjugated with the prostate-specific antigen- (PSA-) specific antibodies to diagnose prostate cancers [138]. In this system, the magnetic microparticles conjugated with PSA-specific antibodies were used to extract the traceable amounts of PSA in the serum samples from patients, and the gold nanoparticles with PSA-specific antibodies and short DNA sequences (the barcodes) were attached to detect this analyte for *in vitro* barcode assay.

### 4.5. Carbon-Based Nanoplatforms—Carbon Nanotubes and Graphene

Graphite is one of the carbon-based natural materials that are widely used in large-scale industrial applications such as steelmaking and battery electrodes [139]. From graphite, carbon-based nanobiomaterials have been engineered with the deeper nanofabrication techniques, which include carbon nanotubes and graphene [140]. These nanobiomaterials are currently and widely regarded as highly attractive biomedical application systems that have a multifunctional nature. In addition, they are incorporated into the conventional existing nanobiomaterials, so-called the hybrid system with further function [141, 142].

Carbon nanotubes are cylindrical nanostructure-based carbon materials, which are synthesized by an arc discharge or chemical vapor deposition of graphite [143]. They are shaped like rolling sheets of carbon into the hollow tubes. Their sizes are ultrasmall, 0.4 to 2 nm in diameter of single-walled carbon nanotubes (SWCNTs) [144, 145]. This system can also be modified to be suitable for biological applications with an addition of functional groups, targeting molecules, and polymers, and so forth to enhance the solubility and biocompatibility [146]. Ruggiero et al. studied the biodistribution and glomerular filtration in the kidney of SWCNT with fluorescence label. This system was applied to the near infrared (NIR) fluorescence imaging and dynamic positron emission tomography (PET) imaging [147]. For the enhancement of solubility of the carbon nanotubes, Liu et al. reported the water soluble carbon nanotubes functionalized with PEG [148]. In addition, this water soluble carbon nanotube system with radio labels and RGD peptide targeted integrins *α*_v_ *β*_3_ for the treatment of human glioblastoma and human colorectal cancer in the U87MG and HT-29 tumor xenograft models. They checked the biodistribution of this system using PET scan in the murine tumor model after intravenous injection.

Graphene is a single planar sheet structured carbon-based material, which is a single atomic plane of graphite, in a honey comb crystal lattice [149]. It is isolated by a simple method for extracting graphene from graphite via exfoliation [150]. Graphene is similar to carbon nanotubes in that it has similar electrical, optical, and thermal properties, although the structure of graphene is two-dimensional atomic sheet different from carbon nanotubes [151]. Graphene can be an attractive material since it has the possibility of being engineered to be structurally thin and flexible. For the biomedical applications, graphene oxide and reduced graphene oxide are more commonly used due to its solubility in aqueous environments and capability of chemical functionalization [152]. Graphene oxide was produced by the oxidation of graphite under acidic conditions (e.g., the modified Hummer's method), and reduced graphene oxide was provided from a reduction of graphene oxide with several reducing reagents (e.g., hydrazine) [153]. They are applied to the biomedical nanomedicine including injectable drug delivery systems for anticancer therapy as carbon nanotubes [154]. For example, the nanographene sheet (NGS) for photothermal therapy (PTT) was reported by Shi et al. [155], which was a six-armed PEG-NGS of 10–50 nm in size with fluorescence labeling for PTT of cancers in the models of 4T1 bearing Balb/c mice, as well as KB and U87MG xenograft models after intravenous injection. In addition, reduced graphene oxide was applied to PTT in tumor models by Yang et al. [156] who used the reduced graphene oxide conjugated with the chimeric form of anti-CD105 mAb, TRC 105, to target and detect the tumor vasculature in the living mice as a theranostic agent.

### 4.6. Dendrimers

Dendrimers are three-dimensional spherical-shaped nanobiomaterials with repeated branches of dendron which contains a single chemical group, called a focal point [157, 158]. The structure of dendrimers consists of a core of initiator, repeated branching units, terminal functional groups, and void spaces, which are rooms for molecular cargo [159]. In the globular and nanosized structures of dendrimers, the terminal functional groups of the outer surface are essential in determining the properties of dendritic macromolecules, which can be interacted and conjugated with other molecules to target the cancer cells and tissues. The commonly used dendrimers in nanomedicines are polyamidoamines (PAMAM) [160], poly(L-lysine) scaffold dendrimers (PLL) [161], polyesters (PGLSA-OH) [162], polypropylimines (PPI) [163], and poly(2,2-bis(hydroxymethyl) propionic acid scaffold dendrimers (bis-MPA) [164]. Some of them are commercially available such as PAMAM dendrimers (Starburst) and PPI dendrimers (Astramol) [165, 166]. Dendrimers can be applied to cancer targeting conjugated with the targeting ligands such as folate, transferrin, antibodies, peptides, and aptamers [167]. In addition, multifunctionality of dendrimers can be a major advantage based on an incorporation of anticancer therapeutics as well as imaging agents [158, 165, 168].

### 4.7. Virus-Based Nanoplatforms (Phage System)

Virus-based nanomaterials have been dramatically investigated in recent years [169]. Viruses are biochemical complexes composed of genomic and proteomic materials. The desired functions of materials for bionanomedicine can be engineered by designing the shape and size of nanoparticles as well as the specific sequence of DNA and proteins. The self-assembled viral architecture can occur in a wide range of shapes and sizes [170] and can offer remarkable structural features of virus that make them excellent candidates for bionanomedicine [171, 172]. Advancements in nanoscale biological engineering of viral particles provide a development of novel pathways to develop nanomedicine for cancer therapeutics. Various types of viruses, such as adenovirus, adeno-associated virus, and bacteriophages, have been utilized for targeted cancer therapy and imaging through genetic and chemical modifications of the virus [173–175].

A bacteriophage (phage) is a prokaryotic virus that can infect the bacterial host cells exploiting the host's biosynthetic machinery to produce many identical copies of the phage itself [176]. There are many types of phages with different genomic materials, replication processes, and shapes such as linear (M13, Fd, and F1) [177] or spherical (MS2) [178]. Some shapes are quite sophisticated; for example, T4 and T7 phages possess an icosahedral head and a long tail connected through a cylindrical body [170]. Over the last two decades, the biochemical landscape of the phage structure has been greatly expanded through genetic engineering [179–182] and site-specific organic synthesis approaches [183–186]. Through genetic engineering, many foreign or synthetic DNAs have been integrated into the phage genome and expressed at various sites of the phage body [182, 187]. Several groups are investigating the engineered filamentous bacteriophage for *in vivo* screening via phage display within organs [188] or cancerous tissue [189, 190], for the purpose of targeted drug delivery [191, 192] or as an imaging agent [193]. For gene delivery applications, therapeutic genetic material can be incorporated into the phage DNA and carried into the cells following a receptor uptake [194]. The phage can be locally targeted to the cell receptors by incorporation of specific targeting and/or internalization peptides (i.e., via RGD or other ligands). To make the phage even more effective than the DNA delivery vehicles, phage can be further decorated with peptides that facilitate endosomal escape or nuclear localization motifs that target the nuclear envelope [195]. The most widely used bacteriophages for gene delivery are M13 filamentous phages [173, 196, 197] and lambda phages [198]. To enhance gene delivery efficiency, the phage with the multifunctional peptides can be produced using a phagemid system, which facilitates manipulation of expressed proteins on viral vectors [197]. Phage display technology has allowed for identification of novel homing peptides that target the unknown cell surface proteins. The targeting peptides can be incorporated into the phage coat proteins through genetic engineering techniques or chemical modifications to improve targeting efficiency [194]. These include peptides (RGD, glioma-binding peptide) [197, 199], HER2 receptor targeting antibody [173], growth factors (EGF and FGF2) [200, 201], and the penton base of adenovirus [198]. Hajitou et al. constructed a hybrid phage with two genes from the phage and nucleus integrating gene from an adeno-associated virus (AAV), called inverted terminal repeats [194]. Although eukaryotic viruses such as AAV have fantastic transgene delivery capabilities, they require an elimination of the native tropism for mammalian cells. In contrast, M13 phages have no tropism for mammalian cells; however, their gene delivery efficiency is poor. Thus, there has been an effort to combine the advantageous aspects of AAV and M13 phages into a single system [194]. This phage displayed integrin-binding peptides (cyclic RGD) on the minor coat proteins and carried the herpes simplex virus thymidine kinase gene (HSVtk). The resulting AAV/phage system provided a superior tumor transduction over the phage alone and was used as the PET imaging agent with [^18^F]FDG and [^18^F]FEAU as well as cancer therapeutics with ganciclovir treatment (Table 1). Ghosh et al. investigated the selective tumor targeting phage-based material for *in vivo* imaging for prostate cancer, acidic and rich in cysteine (SPARC), which is upregulated in various cancers and correlated with poor prognosis [202]. The capsid organization of M13 spatially separates the targeting and imaging moieties. Using p3 to display the targeting ligands (SPARC-binding peptide, SPPTGIN) while assembling multiple magnetic iron oxide nanoparticles (MNPs) along the p8 capsid achieves effective targeting and delivers a larger payload of MNPs per SPARC compared with directly functionalized nanoparticles (Table 1). Compared with nanoparticles that are directly functionalized with targeting peptides, this approach improves the contrast because each SPARC-targeting molecule delivers a larger number of nanoparticles into the cells. Moreover, the targeting ligand and nanoparticles could be easily exchanged for others, making this platform attractive for *in vivo* screening and molecular detection (Table 1).

## 5. Applications of Targeted Delivery Systems from Cells to Clinics

Nanoplatforms generally have the potential to be applied as cancer diagnosis, imaging, and treatment *in vitro* and *in vivo*. Targeted delivery strategies of nanoplatforms are special formulations and carriers with anticancer drugs such as pegylated liposomes, polymeric nanoparticles/micelles, and albumin-based drug carriers. These nanoplatforms that we mentioned above can be applicable to the biomedical approaches such as cancer diagnostics [145], anticancer therapy [203], nanoimaging [204], bimodal imaging [205, 206], and real-time intraoperative imaging [26]. The FDA has approved clinical use of a significant number of anticancer drug products in the nanometer size range including the applications in Table 3. In particular, nanoimaging has become based on the diagnostic potential of an earlier detection in the cancer and other human diseases [207]. In the case of bimodal contrast imaging, bimodal contrast agents allow the assessment of regions of interest using two independent imaging modalities such as MRI reagents and fluorescent agents. In the case of MRI and fluorescence imaging, MRI has an excellent spatial resolution, and fluorescence imaging compensates for the sensitivity of MRI overcoming the limitations of a single-modality imaging [208]. For intraoperative fluorescence imaging, the first human trial in advanced-stage ovarian cancer proceeded with tumor-specific folate receptor-*α* targeted fluorescent agent [26]. This study offers the potential application of intraoperative staging with tumor-specific fluorescence imaging in patients with ovarian cancer by folate receptor-*α* overexpression.

## 6. Conclusion

Human genomic map has accelerated the current biomedical application for the improvement of human healthcare and future therapies encompassing a full understanding of the gene function. Increasing information regarding the gene regulation process will provide the fundamental knowledge for the development of novel therapy in the disease state of cancer. For this streamline of biomedical research, nanoplatforms can take the center stage of participating in the development of targeted nanomedicine for anticancer therapy in the foreseeable future. Using these nanoplatform techniques, targeted anticancer therapy of nanomedicine without toxicity will be able to detect, confirm, and treat various types of cancers as a part of personalized medicine. This therapeutic potential will require more approaches to develop ideal targeted nanoplatforms overcoming toxicity and enhancing biocompatibility, as well as multifunctionality of nanoplatforms. We believe that these cancer targeting studies of nanoplatforms can contribute to the scientific achievement of nanotechnology and nanoplatforms for the development of targeted anticancer therapeutics.

## Figures and Tables

**Table 1:** Recently developed nanoplatforms for cancer targeting in preclinical studies.

Nanoplatforms	Drug	Average size (nm)	Ligand/target	Descriptions	Possible applications	References
Liposomes	Doxorubicin	108	Cyclic Asn-Gly-Arg (cNGR) peptides/CD13 and aminopeptidase N	cNGR decorated, lysolipid-containing temperature sensitive liposomes	Human fibrosarcoma (HT-1080), *in vitro* binding study	[95]

Liposomes inorganic nanoparticles (gold nanorods, ironoxide nanoworms)	Doxorubicin	55	A truncated, tumor-targeted human protein tissue factor (tTF RGD) to induce coagulation on binding to angiogenic *α*_ν_ *β*_3_ receptors to heat specifically directed coagulation-cascade activation in tumours	“Signalling” modules (PEG-decorated gold nanorods, tumor-targeted truncated tissue factor proteins) that target tumors and then locally activate the coagulation cascade to broadcast tumor location to clot-targeted “receiving” nanoparticles in circulation that carry a diagnostic or therapeutic cargo (ironoxide nanoworms, doxorubicin-loaded liposomes)	*In vivo* MDA-MB-435 xenograft tumors, i.v. injection	[97]

Liposomes carbon nanotubes	Paclitaxel	164	anti-ErBb2 (Her2) mAb	Each nanohorn encapsulated within one immunoliposome formulated with PEG and thermally stable and pH sensitive phospholipids for pH sensitive and prolonged release of paclitaxel	SK-BR-3 and BT-20 breast cancer cells, *in vitro* cytotoxicity	[98]

Polymeric nanoparticles	siRNA	80 × 320	—	Cationic lipid-coated PLGA nanoparticles, a unique soft lithography particle molding process (particle replication in nonwetting templates, PRINT)	*In vitro* prostate cancer cell lines	[108]

Polymeric micelles	1,2-Diaminocyclohexane- platinum(II) (DACHPt) (the parent complex of oxaliplatin)	30	—	Long-circulating, drug-loaded polymeric micelles enhancing tumour permeability with a TGF*β* inhibitor	Poorly permeable pancreatic tumors in the mice model (C26 or BxPC3 tumors), i.v. injection	[209]

Nanoconjugates	Splice-switching oligonucleotides (SSOs) (phosphorodiamidate morpholino oligomer (PMO))	13	RGD/integrin *α*_ν_ *β*_3_, a cell surface glycoprotein that is preferentially expressed in angiogenic endothelia	Albumin-based nanoconjugates with fluorescence labeling which are small, highly specific, and noncytotoxic	Tumor spheroids of A375/GFP cell, tumor spheroid study using A375/GFP cells to confirm the uptake and penetration of nanoconjugates in three-dimensional (3D) culture	[128]
	Paclitaxel	2-3	ultrasmall hyaluronic acid (HA)/CD44	Paclitaxel-ultrasmall HA (3–5 kDa) nanoconjugates internalized via CD44 receptor-mediated endocytosis, which allowed paclitaxel to bypass the P-glycoprotein- mediated efflux on the surface of cancer cells	Brain metastasis of breast cancer using MDA-MB-231Br breast cancer cells, i.v. injection	[129]

Gold nanoparticles	Chemiresistors based on functionalized gold nanoparticles in combination with pattern recognition methods	5	—	A sensor array for breath testing of exhaled breath in lung cancer patients	A noninvasive diagnostic tool for lung cancer, *in vitro* array	[137]

Gold nanoparticles magnetic microparticles	An assay for PSA detection with gold nanoparticles based on the PSA-specific Abs and short DNA sequences (barcodes)	30	Prostate-specific antigen (PSA)	Magnetic microparticles conjugated with PSA-specific antibodies to extract trace amounts of the target analyte (PSA) in serum samples from patients and gold nanoparticles with PSA-specific antibodies and short DNA sequences (the barcodes) attached to detect this analyte	An ultrasensitive assay for prostate cancer, *in vitro* barcode array	[138]

Carbon nanotube	Single-walled carbon nanotube (SWCNT) with fluorescence labelling	200–300	—	Biodistribution and examination of glomerular filtration in kidney	NIR fluorescence imaging, dynamic PET imaging, *in vitro *(HK-2 cells at the transwell), *in vivo* (mice, NCr/nu/nu), i.v. injection	[147]
	Water soluble carbon nanotubes functionalized with PEG, radio labels, and RGD peptide	1–5 (diameter), 100–300 (length)	RGD/integrins *α*_ν_ *β*_3_	RGD-pegylated SWCNTs, radio-labelled SWCNTs	U87MG human glioblastoma and HT-29 human colorectal cancer cell lines, U87MG and HT-29 tumour xenograft models for biodistribution (PET), i.v. injection	[148]

Graphene	Nanographene sheet (NGS) for photothermal therapy (PTT)	10–50	—	Six-armed PEG-NGS with fluorescent labeling	4T1 bearing Balb/c mice, KB and U87MG xenograft models, PTT, i.v. injection	[155]
	Reduced graphene oxide (RGO) for PTT	20–80	Human/murine chimeric IgG1 mAb (TRC105)/both human and murine CD105	RGO conjugated to the anti-CD105 antibody TRC105	Tumor vasculature targeting and imaging in living mice as a theranostic agent, *in vitro* (4T1 murine breast cancer, MCF-7 human breast cancer, and human umbilical vein endothelial cells (HUVECs)), *in vivo* (4T1 breast cancer model), i.v. injection	[156]

Phage	Imaging and therapy HSVtk + ganciclovir + [^18^F]FDG and [^18^F]FEAU	7 × 1,000	RGD4C/integrins	Hybrid vector with adeno-associated virus (AAV) and fd-tet derived bacteriophage [AAV/phage (AAVP)] RGD-4C AAVP-HSVtk (herpes simplex virus thymidine kinase)	KS1767 cells RGD-4C AAVP-HSVtk PET images with [^18^F]FDG and [^18^F]FEAU obtained before and after ganciclovir treatment	[194]
	Magnetic iron oxide [(maghemite (*γ*-Fe_2_O_3_) or magnetite (Fe_3_O_4_)] for MRI	6.6 × 880	Acidic and rich in cysteine (SPARC)	SPARC-binding peptide (SPPTGIN) and triglutamate displayed on p3 and p8 coat proteins of M13 phage, respectively	Tumor targeting against prostate cancer	[202]

mAb: monoclonal antibody; PEG: polyethylene glycol; PLGA: poly(lactic-co-glycolic) acid; RGD, arginine, glycine, and aspartic acid (Arg-Gly-Asp).

**Table 2:** Nanomedicines in clinical trials.

Nanoplatforms	Drug	Current status	Mode of delivery*	Ligand/target	Average size (nm)	Descriptions	Types of cancer
Liposomes	Doxorubicin (Myocet)	Phase III	NT	—	100–230	No IRRs, high response, and reduced cardiotoxicity	Kaposi's sarcoma and metastatic breast cancer (phase I/II)
	Vincristine sulfate (Marqibo)	FDA approved (Ph-adult ALL), Phase II (NHL), Phase I (pediatric ALL)	NT	—	115	Extended PK	Acute lymphocytic leukemia (ALL) and non-Hodgkin's lymphoma (NHL)
	Cytarabine Daunorubicin (CPX-351)	Phase III	NT	—	100	Extended PK, high response	Acute myeloid leukemia (AML)
	Paclitaxel (EndoTAG-1)	Phase III	NT	—	160–180	Cationic liposomal formulation	Various solid tumors
	Lurtotecan (OSI-211/NX211)	Phase II completed	NT	—	150	Reduced myelosuppression and high response	Ovarian cancer
	Paclitaxel (LEP-ETU)	Phase II completed	NT	—	150	No IRRs and high response	Metastatic breast cancer
	Camptothecin (S-CKD-602)	Phase I/II	NT	—	100	Pegylated liposomal camptothecin and extended PK	Advanced solid tumors
	Oxaliplatin (MBP-426)	Phase I/II	T	transferrin/transferrin receptor	180	Extended PK	Advanced/metastatic solid tumors
	Doxorubicin (MCC-465)	Phase I (discontinued)	T	F(ab′)_2_ fragment of human mAb GAH or tumor-specific antigen	140	No hand-foot syndrome or cardiotoxicity	Metastatic stomach cancer
	p53 gene (SGT53-01)	Phase Ib	T	scFv/transferrin receptor	90	Improved response	Solid tumors
	RB94 plasmid DNA (SGT-94)	Phase I	T	scFv/transferrin receptor	108	Improved response	Solid tumors
	Doxorubicin (MM-302)	Phase I	T	scFv/ErbB2 (HER2)	75–110		Advanced breast cancer
	Melanoma antigens and IFN*γ* (Lipovaxin-MM)	Phase I	T	Single domain antibody (dAb) fragment (VH)/DC-SIGN		Stable and safe	Melanoma vaccine

Polymers	Paclitaxel (Genexol-PM)	Approved (Korea); phase II/III (USA)	NT	—	<50	Polymeric micelles, increased paclitaxel MTD, high response	Metastatic breast cancer and urothelial carcinoma
	Paclitaxel (NK105)	Phase III	NT	—	85	Core-shell-type polymeric micelles, extended PK, high response, and reduced hypersensitivity	Metastatic/recurrent breast cancer

Polymers	Doxorubicin (SP1049C)	Phase I completed	NT	—	30	Polymeric micelles, high response, and no hand-foot syndrome	Advanced adenocarcinoma of esophagus and gastroesophageal system
	Doxorubicin (NK911)	Phase II (Asia)	NT	—	40	Polymeric micelles, extended PK	Metastatic/recurrent solid tumors
	Paclitaxel-poliglumex (Opaxio)	Phase III	NT	—	10–150	Polymeric micelles, extended PK	Ovarian cancer
	Campthotecin (CRLX101)	Phase Ib/IIa	NT	—	20–50	A pH-sensitive polymer nanocarrier releasing camptothecin in the acidic environment of cancer cells, extended PK, and high response	Advanced solid tumors
	Irinotecan (SN-38, NK012)	Phase I/II	NT	—	20	Polymeric micelle-based active metabolites of camptothecin derivative and extended PK	Solid tumors
	Cisplatin (NC-6004, Nanoplatin)	Phase I/II (Asia)	NT	—	30	Polymeric micelles and extended PK (with the aim of reducing kidney toxicity compared with cisplatin alone)	Advanced/metastatic pancreatic cancer (evaluating nanoplatin in combination with gemcitabine in patients with advanced or metastatic pancreatic cancer)
	Docetaxel (BIND-014)	Phase I completed	T	Peptide/PSMA (solid or metastatic prostate cancer cells by binding to prostate-specific membrane antigen)	100	Enhanced therapeutic efficacy and partial response	Solid tumors
	RRM2 siRNA (CALAA-01)	Phase I	T	Transferrin/transferrin receptor	70	Cyclodextrin-based nanoparticle containing anti-RRM2 siRNA and no DLTs	Solid tumors

PK: pharmacokinetics; DRR: drug release rate; IRRs: infusion-related reactions; Ph-: Philadelphia Chromosome Negative; MTD: maximum tolerable dose; DLTs: dose-limiting toxicities; DC: dendritic cell.

*Mode of delivery: NT: nontargeted (passive targeting), T: targeted (active targeting).

**Table 3:** FDA approved anticancer drugs.

Nanoplatforms	Drug	Company	Properties	Indications	Routes of administration
Liposomes	Abelcet	Enzon	Liposomal amphotericin B	Fungal infections	i.v.
	AmBisome	Gilead Sciences	Liposomal amphotericin B	Fungal and protozoal infections	i.v.
	Amphotec	Three Rivers Pharmaceuticals	Cholesteryl sulfate-based amphotericin B	Fungal infections	i.v.
	DepoCyt	SkyePharma	Liposomal cytarabine	Malignant lymphomatous meningitis	i.t.
	DaunoXome	Gilead Sciences	Liposomal daunorubicin	HIV-related Kaposi's sarcoma	i.v.
	Doxil/Caelyx	Ortho Biotech, Schering-Plough	Liposome-PEG doxorubicin	HIV-related Kaposi's sarcoma, metastatic breast cancer, and metastatic ovarian cancer	i.m.
	Myocet	Zeneus	Liposomal doxorubicin	Combination therapy with cyclophosphamide in metastatic breast cancer	i.v.
	Visudyne	QLT, Novartis	Liposomal verteporfin	Photodynamic therapy (PDT) for age-related macular degeneration, pathologic myopia, and ocular histoplasmosis	i.v.

Polymers	Adagen	Enzon	PEG-adenosine deaminase	Severe combined immunodeficiency disease associated with ADA deficiency	i.m.
	Copaxone	TEVA Pharmaceuticals	L-Glutamic acid, L-alanine, L-lysine, and L-tyrosine copolymer	Multiple sclerosis	s.c.
	Genexol-PM	Samyang	Methoxy-PEG-poly(D, L-lactide) paclitaxel	Metastatic breast cancer	i.v.
	Macugen	OSI Pharmaceuticals	PEG-anti-VEGF aptamer	Age-related macular degeneration	i.r.
	Neulasta	Amgen	PEG-GCSF	Neutropenia associated with cancer Chemotherapy	s.c.
	Oncaspar	Enzon	Pegaspargase (PEG-L-asparaginase)	Acute lymphoblastic leukemia	i.v., i.m.
	Renagel	Genzyme	Poly(allylamine hydrochloride)	End-stage renal disease	Oral
	Somavert	Nektar, Pfizer	PEG-HGF	Acromegaly	s.c.

Others	Abraxane	Abraxis BioScience, AstraZeneca	Albumin-bound paclitaxel	Metastatic breast cancer	i.v.
	Estrasorb	Novavax	Estradiol emulsion	Vasomotot symptoms associated with menopause	Topical and transdermal
	Emend	Elan, Merck	Nanocrystalline aprepitant	Antiemetic	Oral
	Megace ES	Strativa Pharmaceuticals, subsidiary of Par Pharmaceutical. Inc.	Nanocrystalline megestrol acetate	Anorexia, cachexia, or an unexplained significant weight loss in AIDS patients	Oral
	Rapamune	Elan, Wyeth Pharmaceuticals	Nanocrystalline sirolimus	Immunosuppressant	Oral
	TriCor	Elan, Abbott	Nanocrystalline fenofibrate	Antihyperlipidemic	Oral
	Feridex	Bayer Healthcare Pharmaceuticals	Femmoxides solution (superparamagnetic iron oxide)	MRI contrast agent	i.v.

ADA: adenosine deaminase; GCSF: granulocyte colony-stimulating factor; HGF: hepatocyte growth factor; HIV: human immunodeficiency virus; i.m.: intramuscular; i.r.: intravitreous; i.t.: intrathecal; i.v.: intravenous; PEG: polyethyleneglycol; s.c.: subcutaneous; VEGF: vascular endothelial growth factor.

## References

[1] American Cancer Society (2013). *Cancer Facts and Figures 2013*.

[2] Svenson S (2012). Clinical translation of nanomedicines. *Current Opinion in Solid State and Materials Science*.

[3] Mohs AM, Provenzale JM (2010). Applications of nanotechnology to imaging and therapy of brain tumors. *Neuroimaging Clinics of North America*.

[4] Koo Y-EL, Reddy GR, Bhojani M (2006). Brain cancer diagnosis and therapy with nanoplatforms. *Advanced Drug Delivery Reviews*.

[5] National Nanotechnology Initiative (NNI) What is nanotechnology?. http://www.nano.gov/.

[6] Choi HS, Frangioni JV (2010). Nanoparticles for biomedical imaging: fundamentals of clinical translation. *Molecular Imaging*.

[7] Shi J, Votruba AR, Farokhzad OC, Langer R (2010). Nanotechnology in drug delivery and tissue engineering: from discovery to applications. *Nano Letters*.

[8] Zhang L, Gu FX, Chan JM, Wang AZ, Langer RS, Farokhzad OC (2007). Nanoparticles in medicine: therapeutic applications and developments. *Clinical Pharmacology and Therapeutics*.

[9] Lammers T, Hennink WE, Storm G (2008). Tumour-targeted nanomedicines: principles and practice. *British Journal of Cancer*.

[10] Lammers T, Kiessling F, Hennink WE, Storm G (2012). Drug targeting to tumors: principles, pitfalls and (pre-) clinical progress. *Journal of Controlled Release*.

[11] Northfelt DW, Dezube BJ, Thommes JA (1998). Pegylated-liposomal doxorubicin versus doxorubicin, bleomycin, and vincristine in the treatment of AIDS-related Kaposi’s sarcoma: results of a randomized phase III clinical trial. *Journal of Clinical Oncology*.

[12] Strother R, Matei D (2009). Pegylated liposomal doxorubicin in ovarian cancer. *Therapeutics and Clinical Risk Management*.

[13] Bangham AD, Standish MM, Watkins JC (1965). Diffusion of univalent ions across the lamellae of swollen phospholipids. *Journal of Molecular Biology*.

[14] Sen K, Mandal M (2013). Second generation liposomal cancer therapeutics: transition from laboratory to clinic. *International Journal of Pharmaceutics*.

[15] Maeda H, Nakamura H, Fang J (2013). The EPR effect for macromolecular drug delivery to solid tumors: improvement of tumor uptake, lowering of systemic toxicity, and distinct tumor imaging *in vivo*. *Advanced Drug Delivery Reviews*.

[16] Farokhzad OC, Langer R (2009). Impact of nanotechnology on drug delivery. *ACS Nano*.

[17] Zhao M, Yang M, Li X-M (2005). Tumor-targeting bacterial therapy with amino acid auxotrophs of GFP-expressing *Salmonella typhimurium*. *Proceedings of the National Academy of Sciences of the United States of America*.

[18] Kimura NT, Taniguchi S, Aoki K, Baba T (1980). Selective localization and growth of Bifidobacterium bifidum in mouse tumors following intravenous administration. *Cancer Research*.

[19] Matsumura Y, Maeda H (1986). A new concept for macromolecular therapeutics in cancer chemotherapy: mechanism of tumoritropic accumulation of proteins and the antitumor agent smancs. *Cancer Research*.

[20] Fang J, Nakamura H, Maeda H (2011). The EPR effect: unique features of tumor blood vessels for drug delivery, factors involved, and limitations and augmentation of the effect. *Advanced Drug Delivery Reviews*.

[21] Gelderblom H, Verweij J, Nooter K, Sparreboom A (2001). Cremophor EL: the drawbacks and advantages of vehicle selection for drug formulation. *European Journal of Cancer*.

[22] Allen TM, Cullis PR (2004). Drug delivery systems: entering the mainstream. *Science*.

[23] Peer D, Karp JM, Hong S, Farokhzad OC, Margalit R, Langer R (2007). Nanocarriers as an emerging platform for cancer therapy. *Nature Nanotechnology*.

[24] Davis ME, Chen Z, Shin DM (2008). Nanoparticle therapeutics: an emerging treatment modality for cancer. *Nature Reviews Drug Discovery*.

[25] Jain RK, Stylianopoulos T (2010). Delivering nanomedicine to solid tumors. *Nature Reviews Clinical Oncology*.

[26] Van Dam GM, Themelis G, Crane LMA (2011). Intraoperative tumor-specific fluorescence imaging in ovarian cancer by folate receptor-*α* targeting: first in-human results. *Nature Medicine*.

[27] Seymour LW, Ferry DR, Anderson D (2002). Hepatic drug targeting: phase I evaluation of polymer-bound doxorubicin. *Journal of Clinical Oncology*.

[28] Lewis Phillips GD, Li G, Dugger DL (2008). Targeting HER2-positive breast cancer with trastuzumab-DM1, an antibody-cytotoxic drug conjugate. *Cancer Research*.

[29] Qian ZM, Li H, Sun H, Ho K (2002). Targeted drug delivery via the transferrin receptor-mediated endocytosis pathway. *Pharmacological Reviews*.

[30] Gupta B, Levchenko TS, Torchilin VP (2005). Intracellular delivery of large molecules and small particles by cell-penetrating proteins and peptides. *Advanced Drug Delivery Reviews*.

[31] Neri D, Bicknell R (2005). Tumour vascular targeting. *Nature Reviews Cancer*.

[32] Temming K, Schiffelers RM, Molema G, Kok RJ (2005). RGD-based strategies for selective delivery of therapeutics and imaging agents to the tumour vasculature. *Drug Resistance Updates*.

[33] Pasqualini R, Koivunen E, Kain R (2000). Aminopeptidase N is a receptor for tumor-homing peptides and a target for inhibiting angiogenesis. *Cancer Research*.

[34] Matsumura Y, Kataoka K (2009). Preclinical and clinical studies of anticancer agent-incorporating polymer micelles. *Cancer Science*.

[35] Nie S (2010). Editorial: understanding and overcoming major barriers in cancer nanomedicine. *Nanomedicine*.

[36] Perche F, Torchilin VP (2013). Recent trends in multifunctional liposomal nanocarriers for enhanced tumor targeting. *Journal of Drug Delivery*.

[37] Srivastavaa A, ’Connora IBO, Panditb A, Wall JG (2013). Polymer-antibody fragment conjugates for biomedical applications. *Progress in Polymer Science*.

[38] Kintzel PE, Dorr RT (1995). Anticancer drug renal toxicity and elimination: dosing guidelines for altered renal function. *Cancer Treatment Reviews*.

[39] Remesh A (2012). Toxicities of anticancer drugs and its management. *International Journal of Basic & Clinical Pharmacology*.

[40] Jain RK, Stylianopoulos T (2010). Delivering nanomedicine to solid tumors. *Nature Reviews Clinical Oncology*.

[41] Kamaly N, Xiao Z, Valencia PM, Radovic-Moreno AF, Farokhzad OC (2012). Targeted polymeric therapeutic nanoparticles: design, development and clinical translation. *Chemical Society Reviews*.

[42] Julien DC, Behnke S, Wang G, Murdoch GK, Hill RA (2011). Utilization of monoclonal antibody-targeted nanomaterials in the treatment of cancer. *mAbs*.

[43] Kopeček J (2013). Polymer-drug conjugates: origins, progress to date and future directions. *Advanced Drug Delivery Reviews*.

[44] Accardo A, Tesauro D, Morelli G (2013). Peptide-based targeting strategies for simultaneous imaging and therapy with nanovectors. *Polymer Journal *.

[45] Sofou S (2007). Surface-active liposomes for targeted cancer therapy. *Nanomedicine*.

[46] Wang M, Thanou M (2010). Targeting nanoparticles to cancer. *Pharmacological Research*.

[47] Tacar O, Sriamornsak P, Dass CR (2013). Doxorubicin: an update on anticancer molecular action, toxicity and novel drug delivery systems. *Journal of Pharmacy and Pharmacology*.

[48] Volkova M, Russell R (2011). Anthracycline cardiotoxicity: prevalence, pathogenesis and treatment. *Current Cardiology Reviews*.

[49] Krishnan V, Rajasekaran AK (2013). Clinical nanomedicine: a solution to the chemotherapy conundrum in pediatric leukemia therapy. *Clinical Pharmacology and Therapeutics*.

[50] van der Meela R, Vehmeijera LJC, Koka RJ, Storma G, van Gaala EVB (2013). Ligand-targeted particulate nanomedicines undergoing clinical evaluation: current status. *Advanced Drug Delivery Reviews*.

[51] Alakhova DY, Zhao Y, Li S, Kabanov AV (2013). Effect of doxorubicin/pluronic SP1049C on tumorigenicity, aggressiveness, DNA methylation and stem cell markers in murine leukemia. *PloS ONE*.

[52] Matsumura Y, Hamaguchi T, Ura T (2004). Phase I clinical trial and pharmacokinetic evaluation of NK911, a micelle-encapsulated doxorubicin. *British Journal of Cancer*.

[53] Cozzi P, Mongelli N, Suarato A (2004). Recent anticancer cytotoxic agents. *Current Medicinal Chemistry-Anti-Cancer Agents*.

[54] Rowinsky EK, Wright M, Monsarrat B, Donehower RC (1994). Clinical pharmacology and metabolism of Taxol (paclitaxel): update 1993. *Annals of Oncology*.

[55] Rowinsky EK, Donehower RC (1995). Drug therapy: paclitaxel (taxol). *The New England Journal of Medicine*.

[56] Azim HA, Awada A (2012). Clinical development of new formulations of cytotoxics in solid tumors. *Current Opinion in Oncology*.

[57] Fasol U, Frost A, Büchert M (2012). Vascular and pharmacokinetic effects of EndoTAG-1 in patients with advanced cancer and liver metastasis. *Annals of Oncology*.

[58] Koudelka S, Turanek J (2012). Liposomal paclitaxel formulations. *Journal of Controlled Release*.

[59] Kim T-Y, Kim D-W, Chung J-Y (2004). Phase I and pharmacokinetic study of Genexol-PM, a Cremophor-free, polymeric micelle-formulated paclitaxel, in patients with advanced malignancies. *Clinical Cancer Research*.

[60] Negishi T, Koizumi F, Uchino H (2006). NK105, a paclitaxel-incorporating micellar nanoparticle, is a more potent radiosensitising agent compared to free paclitaxel. *British Journal of Cancer*.

[61] Galic VL, Herzog TJ, Wright JD, Lewin SN (2011). Paclitaxel poliglumex for ovarian cancer. *Expert Opinion on Investigational Drugs*.

[62] Hamaguchi T, Matsumura Y, Suzuki M (2005). NK105, a paclitaxel-incorporating micellar nanoparticle formulation, can extend *in vivo* antitumour activity and reduce the neurotoxicity of paclitaxel. *British Journal of Cancer*.

[63] Wang X, Guo Z (2013). Targeting and delivery of platinum-based anticancer drugs. *Chemical Society Reviews*.

[64] Butler JS, Sadler PJ (2013). Targeted delivery of platinum-based anticancer complexes. *Current Opinion in Chemical Biology*.

[65] Zamboni WC (2008). Concept and clinical evaluation of carrier-mediated anticancer agents. *Oncologist*.

[66] Plummer R, Wilson RH, Calvert H (2011). A Phase I clinical study of cisplatin-incorporated polymeric micelles (NC-6004) in patients with solid tumours. *British Journal of Cancer*.

[67] Kümler I, Brünner N, Stenvang J, Balslev E, Nielsen DL (2013). A systematic review on topoisomerase 1 inhibition in the treatment of metastatic breast cancer. *Breast Cancer Research and Treatment*.

[68] Zhang L, Hu Y, Jiang X, Yang C, Lu W, Yang YH (2004). Camptothecin derivative-loaded poly(caprolactone-co-lactide)-b-PEG-b- poly(caprolactone-co-lactide) nanoparticles and their biodistribution in mice. *Journal of Controlled Release*.

[69] Zamboni WC, Ramalingam S, Friedland DM (2009). Phase I and pharmacokinetic study of pegylated liposomal CKD-602 in patients with advanced malignancies. *Clinical Cancer Research*.

[70] Young C, Schluep T, Hwang J, Eliasof S (2011). CRLX101 (formerly IT-101)-A novel nanopharmaceutical of camptothecin in clinical development. *Current Bioactive Compounds*.

[71] Hamaguchi T, Doi T, Eguchi-Nakajima T (2010). Phase I study of NK012, a novel SN-38-incorporating micellar nanoparticle, in adult patients with solid tumors. *Clinical Cancer Research*.

[72] Palmer DH, Young LS, Mautner V (2006). Cancer gene-therapy: clinical trials. *Trends in Biotechnology*.

[73] Xu L, Tang W-H, Huang C-C (2001). Systemic p53 gene therapy of cancer with immunolipoplexes targeted by anti-transferrin receptor scFv. *Molecular Medicine*.

[74] Pirollo KF, Rait A, Zhou Q (2008). Tumor-targeting nanocomplex delivery of novel tumor suppressor RB94 chemosensitizes bladder carcinoma cells *in vitro* and *in vivo*. *Clinical Cancer Research*.

[75] Bartlett DW, Su H, Hildebrandt IJ, Weber WA, Davis ME (2007). Impact of tumor-specific targeting on the biodistribution and efficacy of siRNA nanoparticles measured by multimodality *in vivo* imaging. *Proceedings of the National Academy of Sciences of the United States of America*.

[76] Deshpande PP, Biswas S, Torchilin VP (2013). Current trends in the use of liposomes for tumor targeting. *Nanomedicine*.

[77] Al-Jamal WT, Kostarelos K (2011). Liposomes: from a clinically established drug delivery system to a nanoparticle platform for theranostic nanomedicine. *Accounts of Chemical Research*.

[78] Gregoriadis G (1976). The carrier potential of liposomes in biology and medicine. II. *The New England Journal of Medicine*.

[79] Arias JL (2013). Liposomes in drug delivery: a patent review (2007-present). *Expert Opinion on Therapeutic Patents*.

[80] Preiss MR, Bothun GD (2011). Stimuli-responsive liposome-nanoparticle assemblies. *Expert Opinion on Drug Delivery*.

[81] Manjappa AS, Chaudhari KR, Venkataraju MP (2011). Antibody derivatization and conjugation strategies: application in preparation of stealth immunoliposome to target chemotherapeutics to tumor. *Journal of Controlled Release*.

[82] Zhao Y, Zhang S, Cui S, Wang B, Zhang S (2012). Peptide-based cationic liposome-mediated gene delivery. *Expert Opinion on Drug Delivery*.

[83] Immordino ML, Dosio F, Cattel L (2006). Stealth liposomes: review of the basic science, rationale, and clinical applications, existing and potential. *International Journal of Nanomedicine*.

[84] Mehra NK, Mishra V, Jain NK (2013). Receptor-based targeting of therapeutics. *Therapeutic Delivery*.

[85] Amin M, Badiee A, Jaafari MR Improvement of pharmacokinetic and antitumor activity of PEGylated liposomal doxorubicin by targeting with N-methylated cyclic RGD peptide in mice bearing C-26 colon carcinomas. *International Journal of Pharmaceutics*.

[86] Liu X, Ma S, Dai C (2013). Antiproliferative, antiinvasive, and proapoptotic activity of folate receptor alpha-targeted liposomal doxorubicin in nonfunctional pituitary adenoma cells. *Endocrinology*.

[87] Xiang B, Dong D-W, Shi N-Q (2013). PSA-responsive and PSMA-mediated multifunctional liposomes for targeted therapy of prostate cancer. *Biomaterials*.

[88] http://clinicaltrials.gov/.

[89] Brignole C, Marimpietri D, Pagnan G (2005). Neuroblastoma targeting by c-myb-selective antisense oligonucleotides entrapped in anti-GD2 immunoliposome: immune cell-mediated anti-tumor activities. *Cancer Letters*.

[90] Moret F, Scheglmann D, Reddi E (2013). Folate-targeted PEGylated liposomes improve the selectivity of PDT with meta-tetra(hydroxyphenyl)chlorin (m-THPC). *Photochemical & Photobiological Sciences*.

[91] Zhang Z, Yao J (2012). Preparation of irinotecan-loaded folate-targeted liposome for tumor targeting delivery and its antitumor activity. *AAPS PharmSciTech*.

[92] Yu KF, Zhang WQ, Luo LM (2013). The antitumor activity of a doxorubicin loaded, iRGD-modified sterically-stabilized liposome on B16-F10 melanoma cells: *in vitro* and *in vivo* evaluation. *International Journal of Nanomedicine*.

[93] Wicki A, Rochlitz C, Orleth A (2012). Targeting tumor-associated endothelial cells: Anti-VEGFR2 immunoliposomes mediate tumor vessel disruption and inhibit tumor growth. *Clinical Cancer Research*.

[94] Ma C, Li X, liang X, Jin K, Cao J, Xu W (2013). Novel beta-dicarbonyl derivatives as inhibitors of aminopeptidase N (APN). *Bioorganic & Medicinal Chemistry Letters*.

[95] Negussie AH, Miller JL, Reddy G, Drake SK, Wood BJ, Dreher MR (2010). Synthesis and *in vitro* evaluation of cyclic NGR peptide targeted thermally sensitive liposome. *Journal of Controlled Release*.

[96] Tan S, Li X, Guo Y, Zhang Z (2013). Lipid-enveloped hybrid nanoparticles for drug delivery. *Nanoscale*.

[97] Von Maltzahn G, Park J-H, Lin KY (2011). Nanoparticles that communicate *in vivo* to amplify tumour targeting. *Nature Materials*.

[98] Huang W, Zhang J, Dorn HC, Zhang C (2013). Assembly of bio-nanoparticles for double controlled drug release. *PLoS ONE*.

[99] Karchemski F, Zucker D, Barenholz Y, Regev O (2012). Carbon nanotubes-liposomes conjugate as a platform for drug delivery into cells. *Journal of Controlled Release*.

[100] Narang AS, Chang RK, Hussain MA (2013). Pharmaceutical development and regulatory considerations for nanoparticles and nanoparticulate drug delivery systems. *Journal of Pharmaceutical Sciences*.

[101] Duncan R (2006). Polymer conjugates as anticancer nanomedicines. *Nature Reviews Cancer*.

[102] Banerjee SS, Aher N, Patil R, Khandare J (2012). Poly(ethylene glycol)-prodrug conjugates: concept, design, and applications. *Journal of Drug Delivery*.

[103] Sah H, Thoma LA, Desu HR, Sah E, Wood GC (2013). Concepts and practices used to develop functional PLGA-based nanoparticulate systems. *International Journal of Nanomedicine*.

[104] Danhier F, Ansorena E, Silva JM, Coco R, Le Breton A, Préat V (2012). PLGA-based nanoparticles: an overview of biomedical applications. *Journal of Controlled Release*.

[105] Acharya S, Sahoo SK (2011). PLGA nanoparticles containing various anticancer agents and tumour delivery by EPR effect. *Advanced Drug Delivery Reviews*.

[106] Danhier F, Lecouturier N, Vroman B (2009). Paclitaxel-loaded PEGylated PLGA-based nanoparticles: *in vitro* and *in vivo* evaluation. *Journal of Controlled Release*.

[107] Liang J, Luo Y, Zhao H (2011). Synthetic biology: putting synthesis into biology. *Wiley Interdisciplinary Reviews*.

[108] Hasan W, Chu K, Gullapalli A (2012). Delivery of multiple siRNAs using lipid-coated PLGA nanoparticles for treatment of prostate cancer. *Nano Letters*.

[109] Chittasupho C, Xie S-X, Baoum A, Yakovleva T, Siahaan TJ, Berkland CJ (2009). ICAM-1 targeting of doxorubicin-loaded PLGA nanoparticles to lung epithelial cells. *European Journal of Pharmaceutical Sciences*.

[110] Liang C, Yang Y, Ling Y, Huang Y, Li T, Li X (2011). Improved therapeutic effect of folate-decorated PLGA-PEG nanoparticles for endometrial carcinoma. *Bioorganic and Medicinal Chemistry*.

[111] Zhao H, Yung LYL (2009). Addition of TPGS to folate-conjugated polymer micelles for selective tumor targeting. *Journal of Biomedical Materials Research - Part A*.

[112] Taylor RM, Severns V, Brown DC, Bisoffi M, Sillerud LO (2012). Prostate cancer targeting motifs: Expression of *α*v*β*3, neurotensin receptor 1, prostate specific membrane antigen, and prostate stem cell antigen in human prostate cancer cell lines and xenografts. *Prostate*.

[113] Dhar S, Gu FX, Langer R, Farokhza OC, Lippard SJ (2008). Targeted delivery of cisplatin to prostate cancer cells by aptamer functionalized Pt(IV) prodrug-PLGA - PEG nanoparticles. *Proceedings of the National Academy of Sciences of the United States of America*.

[114] Danhier F, Pourcelle V, Marchand-Brynaert J, Jérôme C, Feron O, Préat V (2012). Targeting of tumor endothelium by RGD-grafted PLGA-nanoparticles. *Methods in Enzymology*.

[115] Hassert R, Hoffmeister PG, Pagel M, Hacker M, Schulz-Siegmund M, Beck-Sickinger AG (2012). On-resin synthesis of an acylated and fluorescence-labeled cyclic integrin ligand for modification of poly(lactic-co-glycolic acid). *Chemistry & Biodiversity*.

[116] Aravind A, Jeyamohan P, Nair R (2012). AS1411 aptamer tagged PLGA-lecithin-PEG nanoparticles for tumor cell targeting and drug delivery. *Biotechnology and Bioengineering*.

[117] Guo J, Gao X, Su L (2011). Aptamer-functionalized PEG-PLGA nanoparticles for enhanced anti-glioma drug delivery. *Biomaterials*.

[118] Fadeel B (2012). Clear and present danger? Engineered nanoparticles and the immune system. *Swiss Medical Weekly*.

[119] Becker R, Dembek C, White LA, Garrison LP (2012). The cost offsets and cost-effectiveness associated with pegylated drugs: a review of the literature. *Expert Review of Pharmacoeconomics & Outcomes Research*.

[120] Kommareddy S, Tiwari SB, Amiji MM (2005). Long-circulating polymeric nanovectors for tumor-selective gene delivery. *Technology in Cancer Research and Treatment*.

[121] Raha S, Paunesku T, Woloschak G (2011). Peptide-mediated cancer targeting of nanoconjugates. *Wiley Interdisciplinary Reviews: Nanomedicine and Nanobiotechnology*.

[122] Segal E, Satchi-Fainaro R (2009). Design and development of polymer conjugates as anti-angiogenic agents. *Advanced Drug Delivery Reviews*.

[123] Xiong Y, Jiang W, Shen Y (2012). A Poly(*γ*, l-glutamic acid)-citric acid based nanoconjugate for cisplatin delivery. *Biomaterials*.

[124] Zhao X, Li H, Lee RJ (2008). Targeted drug delivery via folate receptors. *Expert Opinion on Drug Delivery*.

[125] Zhao XB, Lee RJ (2004). Tumor-selective targeted delivery of genes and antisense oligodeoxyribonucleotides via the folate receptor. *Advanced Drug Delivery Reviews*.

[126] Teng L, Xie J, Lee RJ (2012). Clinical translation of folate receptor-targeted therapeutics. *Expert Opinion on Drug Delivery*.

[127] Zwicke GL, Mansoori GA, Jeffery CJ (2012). Utilizing the folate receptor for active targeting of cancer nanotherapeutics. *Nano Reviews*.

[128] Ming X, Carver K, Wu L (2013). Albumin-based nanoconjugates for targeted delivery of therapeutic oligonucleotides. *Biomaterials*.

[129] Mittapalli RK, Liu X, Adkins CE (2013). Paclitaxel-hyaluronic nanoconjugates prolong overall survival in a preclinical brain metastases of breast cancer model. *Molecular Cancer Therapeutics*.

[130] Boyer C, Whittaker MR, Bulmus V, Liu J, Davis TP (2010). The design and utility of polymer-stabilized iron-oxide nanoparticles for nanomedicine applications. *NPG Asia Materials*.

[131] Polyak B, Friedman G (2009). Magnetic targeting for site-specific drug delivery: Applications and clinical potential. *Expert Opinion on Drug Delivery*.

[132] Meng J, Fan J, Galiana G (2009). LHRH-functionalized superparamagnetic iron oxide nanoparticles for breast cancer targeting and contrast enhancement in MRI. *Materials Science and Engineering C*.

[133] Yu MK, Jeong YY, Park J (2008). Drug-loaded superparamagnetic iron oxide nanoparticles for combined cancer imaging and therapy *in vivo*. *Angewandte Chemie - International Edition*.

[134] Kohler N, Sun C, Wang J, Zhang M (2005). Methotrexate-modified superparamagnetic nanoparticles and their intracellular uptake into human cancer cells. *Langmuir*.

[135] Figuerola A, Di Corato R, Manna L, Pellegrino T (2010). From iron oxide nanoparticles towards advanced iron-based inorganic materials designed for biomedical applications. *Pharmacological Research*.

[136] Shipp G (2006). Ultrasensitive measurement of protein and nucleic acid biomarkers for earlier disease detection and more effective therapies. *Biotechnology Healthcare Journal*.

[137] Peng G, Tisch U, Adams O (2009). Diagnosing lung cancer in exhaled breath using gold nanoparticles. *Nature Nanotechnology*.

[138] Thaxton CS, Elghanian R, Thomas AD (2009). Nanoparticle-based bio-barcode assay redefines “undetectable” PSA and biochemical recurrence after radical prostatectomy. *Proceedings of the National Academy of Sciences of the United States of America*.

[139] Singh V, Joung D, Zhai L, Das S, Khondaker SI, Seal S (2011). Graphene based materials: past, present and future. *Progress in Materials Science*.

[140] Cha C, Shin SR, Annabi N, Dokmeci MR, Khademhosseini A (2013). Carbon-based nanomaterials: multifunctional materials for biomedical engineering. *ACS Nano*.

[141] Biswas A, Bayer IS, Biris AS, Wang T, Dervishi E, Faupel F (2012). Advances in top-down and bottom-up surface nanofabrication: techniques, applications & future prospects. *Advances in Colloid and Interface Science*.

[142] Sharma P, Bhalla V, Prasad ES, Dravid V, Shekhawat G, Suri CR (2012). Enhancing graphene/CNT based electrochemical detection using magneto-nanobioprobes. *Scientific Reports*.

[143] Mallick K, Strydom AM (2013). Biophilic carbon nanotubes. *Colloids and Surfaces B*.

[144] Kateb B, Van Handel M, Zhang L, Bronikowski MJ, Manohara H, Badie B (2007). Internalization of MWCNTs by microglia: possible application in immunotherapy of brain tumors. *NeuroImage*.

[145] Kateb B, Chiu K, Black KL (2011). Nanoplatforms for constructing new approaches to cancer treatment, imaging, and drug delivery: What should be the policy?. *NeuroImage*.

[146] Bianco A, Kostarelos K, Partidos CD, Prato M (2005). Biomedical applications of functionalised carbon nanotubes. *Chemical Communications*.

[147] Ruggiero A, Villa CH, Bander E (2010). Paradoxical glomerular filtration of carbon nanotubes. *Proceedings of the National Academy of Sciences of the United States of America*.

[148] Liu Z, Cai W, He L (2007). *in vivo* biodistribution and highly efficient tumour targeting of carbon nanotubes in mice. *Nature Nanotechnology*.

[149] Chung C, Kim Y-K, Shin D, Ryoo S-R, Hong BH, Min D-H (2013). Biomedical applications of graphene and graphene oxide. *Accounts of Chemical Research*.

[150] Cai M, Thorpe D, Adamsonb DH, Schniepp HC (2012). Methods of graphite exfoliation. *Journal of Materials Chemistry*.

[151] Butler SZ, Hollen SM, Cao L (2013). Progress, challenges, and opportunities in two-dimensional materials beyond graphene. *ACS Nano*.

[152] Shen H, Zhang L, Liu M, Zhang Z (2012). Biomedical applications of graphene. *Theranostics*.

[153] Dreyer DR, Park S, Bielawski CW, Ruoff RS (2010). The chemistry of graphene oxide. *Chemical Society Reviews*.

[154] Yang K, Feng L, Shi X, Liu Z (2013). Nano-graphene in biomedicine: theranostic applications. *Chemical Society Reviews*.

[155] Shi S, Yang K, Hong H (2013). Tumor vasculature targeting and imaging in living mice with reduced graphene oxide. *Biomaterials*.

[156] Yang K, Zhang S, Zhang G, Sun X, Lee S-T, Liu Z (2010). Graphene in mice: ultrahigh *in vivo* tumor uptake and efficient photothermal therapy. *Nano Letters*.

[157] Svenson S, Tomalia DA (2005). Dendrimers in biomedical applications: reflections on the field. *Advanced Drug Delivery Reviews*.

[158] Menjoge AR, Kannan RM, Tomalia DA (2010). Dendrimer-based drug and imaging conjugates: design considerations for nanomedical applications. *Drug Discovery Today*.

[159] Medina SH, El-Sayed MEH (2009). Dendrimers as carriers for delivery of chemotherapeutic agents. *Chemical Reviews*.

[160] Xu Q, Wang C-H, Pack DW (2010). Polymeric carriers for gene delivery: chitosan and poly(amidoamine) dendrimers. *Current Pharmaceutical Design*.

[161] Huang R, Han L, Li J (2011). Chlorotoxin-modified macromolecular contrast agent for MRI tumor diagnosis. *Biomaterials*.

[162] Morgan MT, Nakanishi Y, Kroll DJ (2006). Dendrimer-encapsulated camptothecins: increased solubility, cellular uptake, and cellular retention affords enhanced anticancer activity *in vitro*. *Cancer Research*.

[163] Wang F, Cai X, Su Y Reducing cytotoxicity while improving anti-cancer drug loading capacity of polypropylenimine dendrimers by surface acetylation. *Acta Biomaterialia*.

[164] Carlmark A, Malmstrom E, Malkoch M (2013). Dendritic architectures based on bis-MPA: functional polymeric scaffolds for application-driven research. *Chemical Society Reviews*.

[165] Svenson S, Chauhan AS (2008). Dendrimers for enhanced drug solubilization. *Nanomedicine*.

[166] Svenson S (2009). Dendrimers as versatile platform in drug delivery applications. *European Journal of Pharmaceutics and Biopharmaceutics*.

[167] Yu B, Tai HC, Xue W, Lee LJ, Lee RJ (2010). Receptor-targeted nanocarriers for therapeutic delivery to cancer. *Molecular Membrane Biology*.

[168] Kesharwani P, Gajbhiye V, Jain NK (2012). A review of nanocarriers for the delivery of small interfering RNA. *Biomaterials*.

[169] Sarikaya M, Tamerler C, Jen AK-Y, Schulten K, Baneyx F (2003). Molecular biomimetics: nanotechnology through biology. *Nature Materials*.

[170] Farr R, Choi DS, Lee SW (2013). Phage-based nanomaterials for biomedical applications. *Acta Biomaterialia*.

[171] Steinmetz NF (2010). Viral nanoparticles as platforms for next-generation therapeutics and imaging devices. *Nanomedicine*.

[172] Plummer EM, Manchester M (2011). Viral nanoparticles and virus-like particles: platforms for contemporary vaccine design. *Wiley Interdisciplinary Reviews*.

[173] Poul M-A, Marks JD (1999). Targeted gene delivery to mammalian cells by filamentous bacteriophage. *Journal of Molecular Biology*.

[174] Frenkel D, Solomon B (2002). Filamentous phage as vector-mediated antibody delivery to the brain. *Proceedings of the National Academy of Sciences of the United States of America*.

[175] Nishimoto T, Yoshida K, Miura Y (2009). Oncolytic virus therapy for pancreatic cancer using the adenovirus library displaying random peptides on the fiber knob. *Gene Therapy*.

[176] Luria SE (1950). Bacteriophage: an essay on virus reproduction. *Science*.

[177] Straus SK, Scott WRP, Symmons MF, Marvin DA (2008). On the structures of filamentous bacteriophage Ff (fd, f1, M13). *European Biophysics Journal*.

[178] Valegard K, Liljas L, Fridborg K, Unge T (1990). The three-dimensional structure of the bacterial virus MS2. *Nature*.

[179] Merzlyak A, Indrakanti S, Lee S-W (2009). Genetically engineered nanofiber-like viruses for tissue regenerating materials. *Nano Letters*.

[180] Parmley SF, Smith GP (1988). Antibody-selectable filamentous fd phage vectors: affinity purification of target genes. *Gene*.

[181] Scott JK, Smith GP (1990). Searching for peptide ligands with an epitope library. *Science*.

[182] Smith GP, Petrenko VA (1997). Phage display. *Chemical Reviews*.

[183] Carrico ZM, Romanini DW, Mehl RA, Francis MB (2008). Oxidative coupling of peptides to a virus capsid containing unnatural amino acids. *Chemical Communications*.

[184] Miller RA, Presley AD, Francis MB (2007). Self-assembling light-harvesting systems from synthetically modified tobacco mosaic virus coat proteins. *Journal of the American Chemical Society*.

[185] Stephanopoulos N, Carrico ZM, Francis MB (2009). Nanoscale integration of sensitizing chromophores and porphyrins with bacteriophage MS2. *Angewandte Chemie*.

[186] Schlick TL, Ding Z, Kovacs EW, Francis MB (2005). Dual-surface modification of the tobacco mosaic virus. *Journal of the American Chemical Society*.

[187] Smith GP (1985). Filamentous fusion phage: novel expression vectors that display cloned antigens on the virion surface. *Science*.

[188] Pasqualini R, Ruoslahti E (1996). Organ targeting *in vivo* using phage display peptide libraries. *Nature*.

[189] Arap W, Pasqualini R, Ruoslahti E (1998). Cancer treatment by targeted drug delivery to tumor vasculature in a mouse model. *Science*.

[190] Krag DN, Shukla GS, Shen G-P (2006). Selection of tumor-binding ligands in cancer patients with phage display libraries. *Cancer Research*.

[191] Dickerson TJ, Janda KD (2005). Recent advances for the treatment of cocaine abuse: central nervous system immunopharmacotherapy. *AAPS Journal*.

[192] Yacoby I, Bar H, Benhar I (2007). Targeted drug-carrying bacteriophages as antibacterial nanomedicines. *Antimicrobial Agents and Chemotherapy*.

[193] Frenkel D, Solomon B (2002). Filamentous phage as vector-mediated antibody delivery to the brain. *Proceedings of the National Academy of Sciences of the United States of America*.

[194] Hajitou A, Trepel M, Lilley CE (2006). A hybrid vector for ligand-directed tumor targeting and molecular imaging. *Cell*.

[195] Martin ME, Rice KG (2007). Peptide-guided gene delivery. *AAPS Journal*.

[196] Larocca D, Witte A, Johnson W, Pierce GF, Baird A (1998). Targeting bacteriophage to mammalian cell surface receptors for gene delivery. *Human Gene Therapy*.

[197] Mount JD, Samoylova TI, Morrison NE, Cox NR, Baker HJ, Petrenko VA (2004). Cell targeted phagemid rescued by preselected landscape phage. *Gene*.

[198] Piersanti S, Cherubini G, Martina Y (2004). Mammalian cell transduction and internalization properties of *λ* phages displaying the full-length adenoviral penton base or its central domain. *Journal of Molecular Medicine*.

[199] Hart SL, Knight AM, Harbottle RP (1994). Cell binding and internalization by filamentous phage displaying a cyclic Arg-Gly-Asp-containing peptide. *Journal of Biological Chemistry*.

[200] Burg MA, Jensen-Pergakes K, Gonzalez AM, Ravey P, Baird A, Larocca D (2002). Enhanced phagemid particle gene transfer in camptothecin-treated carcinoma cells. *Cancer Research*.

[201] Seow Y, Wood MJ (2009). Biological gene delivery vehicles: beyond viral vectors. *Molecular Therapy*.

[202] Ghosh D, Lee Y, Thomas S (2012). M13-templated magnetic nanoparticles for targeted *in vivo* imaging of prostate cancer. *Nature Nanotechnology*.

[203] Muthu MS, Wilson B (2010). Multifunctional radionanomedicine: a novel nanoplatform for cancer imaging and therapy. *Nanomedicine*.

[204] Bulte JW, Modo M (2007). *Nanoparticles in Biomedical Imaging: Emerging Technologies and Applications*.

[205] Janczewski D, Zhang Y, Das GK (2011). Bimodal magnetic-fluorescent probes for bioimaging. *Microscopy Research and Technique*.

[206] Jarzyna PA, Gianella A, Skajaa T (2010). Multifunctional imaging nanoprobes. *Wiley Interdisciplinary Reviews*.

[207] Jeff MMJM, Bulte WM (2008). Nanoparticles in biomedical imaging, emerging technologies and applications. *Fundamental Biomedical Technologies*.

[208] McMahon MT, Gilad AA, DeLiso MA, Cromer Berman SM, Bulte JWM, Van Zijl PCM (2008). New “multicolor” polypeptide diamagnetic chemical exchange saturation transfer (DIACEST) contrast agents for MRI. *Magnetic Resonance in Medicine*.

[209] Cabral H, Matsumoto Y, Mizuno K (2011). Accumulation of sub-100 nm polymeric micelles in poorly permeable tumours depends on size. *Nature Nanotechnology*.

